# Esophageal epidermoid metaplasia: diagnostic challenges and therapeutic strategies

**DOI:** 10.1093/jcag/gwae044

**Published:** 2024-10-24

**Authors:** Robert Bechara, Franco D’Anna, Wang Tao

**Affiliations:** Department of Medicine, Queen’s University, Kingston, Ontario, K7L 2V6, Canada; Faculty of Medicine, McGill University, Montreal, Quebec, H3G 2M1, Canada; Department of Medicine, Queen’s University, Kingston, Ontario, K7L 2V6, Canada

A 58-year-old female smoker underwent pan-endoscopy due to iron deficiency anaemia, during which irregular white plaques were incidentally found throughout her oesophagus using White Light Endoscopy (WLE) and Blue Light Imaging (BLI) (Figure 1A,B). They had a fine nodular appearance on low magnification (Figure 1D,E) and appeared as Lugol voiding lesions when stained with 1.25% Lugol’s iodine (Figure 1C). This staining showed well-demarcated, slightly elevated plaques that could not be scrapped off and, unlike in normal squamous mucosa or typical squamous neoplasia, the microvasculature was not visible.^1^ This was due to keratinisation confirmed via biopsies that also showed a granular layer, mimicking skin epidermis (Figure 1F). No signs of squamous high-grade dysplasia or carcinoma were detected. These findings are consistent with extensive esophageal epidermoid metaplasia (EEM). The patient was enrolled in an endoscopic surveillance program.

**Figure 1.**
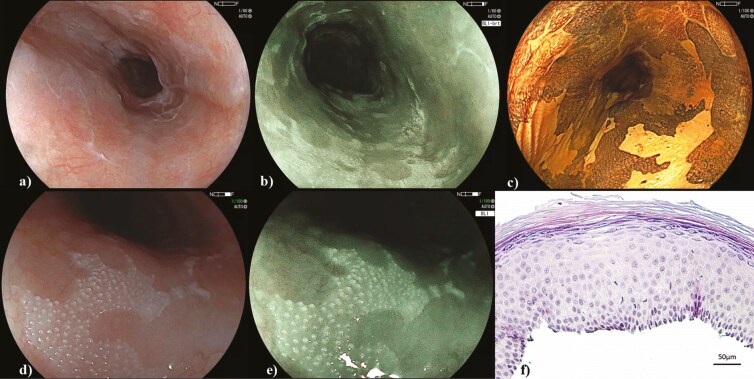
Visualisation of esophageal epidermoid metaplasia (A) White Light Endoscopy (WLE) (B) Blue Light Imaging (BLI) (C) 1.25% Lugol’s (D) low magnification with WLE (E) low magnification with BLI (F) Histology with haematoxylin–eosin–saffron stain at 200× magnification showing abnormal surface keratinisation and presence of a granular layer. Endoscopic images courtesy of Dr Bechara and histologic images courtesy of Dr Tao.

EEM, often located in the middle or distal third of the oesophagus, is described as above but can vary in appearance, sometimes showing granular, cobblestone, or lacy characteristics.^2–5^With subtle findings and non-specific symptoms like reflux and dysphagia, EEM is often underdiagnosed.^2,3^

Patients with EEM often have histories of tobacco and alcohol use, risk factors heavily associated with esophageal squamous cell carcinoma (ESCC).^2,3^ Furthermore, EEM shares genetic mutations with ESCC, notably in TP53, suggesting that EEM may be a precursor to ESCC.^4,5^

Management of EEM without advanced dysplasia remains debated.^5^ Some recommend routine surveillance; others suggest endoscopic mucosal resection for small or dysplastic lesions and radiofrequency ablation for larger ones.^2^ For extensive, non-dysplastic lesions, surveillance is advised every six months with 4-quadrant biopsies every 1–2 cm, transitioning to annual exams if no dysplasia or progression occurs.^2,3,5^

## Supplementary Material

gwae044_Supplementary_Material

## Data Availability

This article includes the use of endoscopic and pathological images from a specific patient case. These images are integral to the discussion and are included within the manuscript. As the article primarily synthesises existing knowledge, no new datasets were generated. The images and data used in this study are available within the article, and any additional inquiries can be directed to the corresponding author, subject to patient confidentiality, and ethical considerations.
